# Role of Uremic Toxins for Kidney, Cardiovascular, and Bone Dysfunction

**DOI:** 10.3390/toxins10050202

**Published:** 2018-05-16

**Authors:** Hideki Fujii, Shunsuke Goto, Masafumi Fukagawa

**Affiliations:** 1 Division of Nephrology and Kidney Center, Kobe University Graduate School of Medicine, Kobe 650-0017, Japan; fhideki@med.kobe-u.ac.jp (H.F.); sgoto@med.kobe-u.ac.jp (S.G.); 2 Division of Nephrology, Endocrinology, and Metabolism, Tokai University School of Medicine, Isehara 259-1193, Japan

**Keywords:** uremic toxins, chronic kidney disease, cardiovascular disease, bone disease, indoxyl sulfate, asymmetric dimethylarginine, *p*-cresylsulfate

## Abstract

With decreasing kidney function, cardiovascular disease (CVD) and mineral bone disorders frequently emerge in patients with chronic kidney disease (CKD). For these patients, in addition to the traditional risk factors, non-traditional CKD-specific risk factors are also associated with such diseases and conditions. One of these non-traditional risk factors is the accumulation of uremic toxins (UTs). In addition, the accumulation of UTs further deteriorates kidney function. Recently, a huge number of UTs have been identified. Although many experimental and clinical studies have reported associations between UTs and the progression of CKD, CVD, and bone disease, these relationships are very complex and have not been fully elucidated. Among the UTs, indoxyl sulfate, asymmetric dimethylarginine, and *p*-cresylsulfate have been of particular focus, up until now. In this review, we summarize the pathophysiological influences of these UTs on the kidney, cardiovascular system, and bone, and discuss the clinical data regarding the harmful effects of these UTs on diseases and conditions.

## 1. Introduction

It is well known that the incidence of cardiovascular events and mortality is much higher in patients with chronic kidney disease (CKD) [1], because such patients, not only have classical risk factors for cardiovascular disease (CVD), such as hypertension, diabetes mellitus, dyslipidemia, and hyperuricemia, etc., but also have many non-classical CKD-specific risk factors for CVD, including anemia, volume over-load, mineral bone disorders, inflammation, malnutrition, and activation of the sympathetic nervous system and renin-angiotensin-aldosterone systems, among others [2]. One such non-classical CKD-specific risk factor is uremic toxins (UTs). Generally speaking, UTs are substances that accumulate in the body following decreased kidney function. Furthermore, the toxicities of UTs have been shown in various experimental studies. UTs are classified into three groups: Small water-soluble solutes, middle molecule, and protein-bound solutes (Table 1) [3,4,5]. As the characteristics suggest, UTs play a crucial role in the progression of CKD and CVD.

Although various UTs are undoubtedly involved in the progression of multiple organ damage, the focus of the present review is on the roles of indoxyl sulfate (IS), asymmetric dimethylarginine (ADMA), and *p*-cresylsulfate (pCS) in the pathogenesis of the progression of CKD, CVD, and bone abnormalities.

## 2. Characteristics of IS, ADMA, and pCS

IS, a naturally occurring tryptophan metabolite, is a uremic toxin [6]. IS is produced in the liver from indole, a tryptophan derivative that is generated by bacteria in the large intestine and binds efficiently to albumin in the blood.

ADMA is an endogenous competitive inhibitor of nitric oxide (NO) synthase. Therefore, ADMA decreases production of NO, which is a potent anti-atherosclerotic molecule [7], and thereby leads to progressive damage due to impaired vascular function in the kidneys, heart, and other organs. Furthermore, it has been suggested that serum ADMA levels are a surrogate marker of endothelial dysfunction and/or incipient atherosclerosis.

pCS is also a protein-bound UT generated from *p*-cresol in the intestine. As with IS, *p*-cresol is also produced by intestinal bacteria as a result of the metabolism of tyrosine and phenylalanine [8].

**Figure 1:**
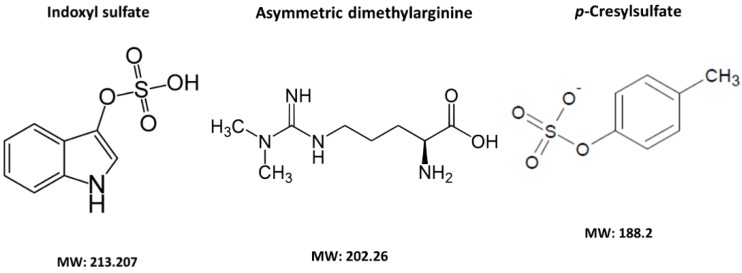
Structure and molecular weight of IS, ADMA, and pCS.

The structure and molecular weight of these three UTs are shown in Figure 1.

## 3. UTs and CKD

### 3.1. Pathophysiological Role of IS in the Progression of CKD

It is generally recognized that serum IS levels increase with declining kidney function, and that the elevation of serum IS levels contributes to the progression of kidney injury [9]. Various pathophysiological mechanisms are involved in this process. An experimental study using a rat model of CKD demonstrated that the administration of IS accelerates glomerular sclerosis, and that combination therapy with dietary protein restriction and administration of AST-120, which is a charcoal adsorbent, could prevent its progression [10]. Therefore, this strategy is appropriate in clinical settings, since IS is synthesized from protein.

Aryl hydrocarbon receptor (AhR) is a ligand-activated transcription factor receptor that is activated by dioxins and some UTs, including IS and kynurenine etc., and thereby interacts with various regulatory and signaling protein [11]. Recent reports demonstrated that AhR activation is associated with vascular inflammation, leukocyte activation, thrombosis, reactive oxygen species, and cardiotoxicity. As well as AhR, epidermal growth factor receptor (EGFR) activation by IS could also contribute to renal tissue remodeling and arteriosclerosis [12,13]. Thus, AhR and EGFR activation are important mechanisms of toxicity of IS.

**Figure 2:**
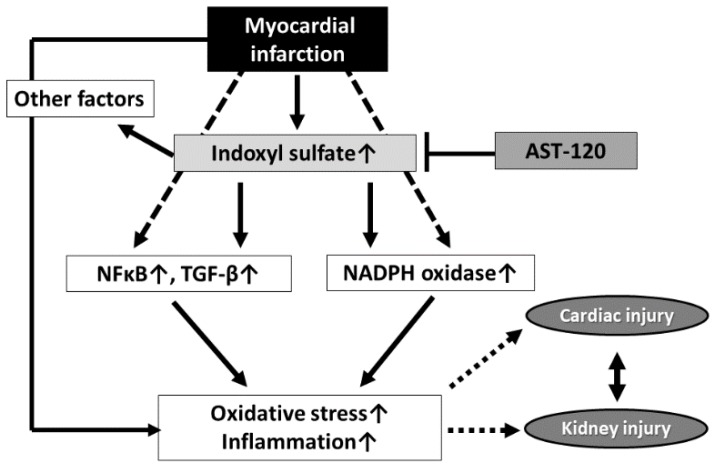
Putative role of IS in the development of cardiorenal syndrome after myocardial infarction.

Furthermore, several detailed mechanisms of the progression of kidney disease have been proposed. For instance, IS accelerates kidney fibrosis by increasing expressions of TGF-β, TIMP-1, and pro collagen I [14]. As a direct effect of IS on kidney cells, IS is taken up by kidney tubular cells via organic anion transporter-1 (OAT-1) and organic anion transporter-3 (OAT-3), and various accumulative effects are exerted in the kidney. IS accumulation in tubular cells destroys the anti-oxidative system, which leads to cellular dysfunction and increased oxidative stress [15]. Moreover, increased PAI-1 expression by activating NF-κB induces dysfunction of kidney tubular cells [16]. Increased expressions of TGF-β, MCP-1, and ICAM-1 also contribute to the progression of kidney damage [17]. Although a decrease in klotho expression is reportedly associated with kidney fibrosis, IS reduces klotho expression in the kidney [18]. IS induces both local and systemic oxidative stress, which leads to systemic organ damage [19]. In fact, lowering serum IS levels by the administration of AST-120 reduces oxidative stress, thereby ameliorating kidney injury [20,21]. Furthermore, recent experimental studies have found that serum IS levels were elevated after myocardial infarction with no decrease in kidney function, while kidney injury markers and oxidative stress were increased in the serum and kidney [22,23]. Treatment with AST-120 decreased the expression of these markers and oxidative stress. Overall, these findings suggest that IS is associated with the pathophysiology of cardiorenal syndrome and that AST-120 could improve these abnormalities (Figure 2).

IS plays a role in the pathophysiology of acute kidney injury by influencing the NO-dependent pathway and decreasing the number of endothelial progenitor cells (EPCs) [24], which delays recovery from kidney injury and leads to CKD.

The association between CKD and the renin-angiotensin system (RAS) is well known, and many clinical studies have demonstrated the effectiveness of RAS inhibitors for the treatment of CKD. IS was found to activate the RAS, which then induces epithelial-to-mesenchymal transition and apoptosis of renal tubular cells [25].

Thus, IS is associated with the progression of kidney injury through the activation of several pathways.

### 3.2. The Effects of IS on Kidney Injury

Clinical studies have also concluded that serum IS levels were significantly and negatively correlated with the glomerular filtration rate (GFR), and namely, that they elevate with declining kidney function [26,27]. From about stage 4 of CKD, serum IS levels rapidly increase.

AST-120 absorbs and decreases the amounts of various UTs, including IS, and is therefore often used for treatment of CKD in clinical settings. It has been reported that AST-120 can reduce oxidative stress in hemodialysis patients [28]. A previous observational study showed that AST-120 administration might delay the progression of CKD and reduce mortality [29,30]. Moreover, a randomized controlled trial (RCT) in Japan demonstrated that AST-120 administration significantly ameliorated the decline in GFR in CKD patients [31]. On the other hand, the results of two international RCTs (EPPIC-1 and EPPIC-2) [32] and a Korean RCT (K-STAR) [33] could not confirm the effectiveness of AST-120 on the preservation of kidney function. However, the results of the EPPIC-2 trial showed that the declining GFR was significantly lower in the AST-120 treatment group and post hoc analyses of patients in the United States who participated in the EPPIC-1 trial demonstrated that AST-120 significantly prevents the progression of CKD [34]. In addition, post hoc analysis of the K-STAR trial also revealed that AST-120 treatment was more protective of kidney function, especially in diabetic patients with CKD, and that it was more effective in the prevention of CVD [35].

Possible explanations of why these RCTs failed to demonstrate the effectiveness of AST-120 on CKD include the study designs, the prescribed doses of AST-120, the lengths of the observational periods, and differences in the clinical practices among the enrolled countries. To ascertain the usefulness of AST-120 for CKD patients, well-designed and large scale RCTs are needed.

### 3.3. Pathophysiological Role of ADMA in the Progression of CKD

Although plasma ADMA levels increase with declining kidney function, this elevation is not merely due to impaired kidney clearance of ADMA. Reportedly, more than 90% of ADMA is metabolized by dimethylarginine–dimethylaminohydrolase (DDAH)-I and II, which are highly expressed in the kidney [36]. DDAH expression is decreased in CKD, which contributes to the elevation of plasma ADMA levels [37]. Although it is well known that ADMA is a key modulator of NO production, NO is also a crucial factor for the progression of CKD. Altering the bioavailability of NO by ADMA not only affects blood pressure, but also regulates the function of the glomerular filtration barrier and is associated with albumin permeability [38].

The roles of ADMA in the progression of diabetic kidney disease have also been investigated. Our previous study demonstrated that elevated serum ADMA levels and decreased intrarenal expressions of DDAH-I and II were observed in diabetic model rats [39]. In fact, urinary excretion of protein was decreased by suppressing the elevation of ADMA levels in CKD and diabetic model rats [40,41].

Chronic ischemia and hypoxia in the tubulointerstitium by ADMA, leads to tubulointerstitial fibrosis and peritubular capillary loss, which are important pathophysiological changes in the progression of CKD. Elevation of plasma ADMA levels increases apoptosis and inhibits proliferation and migration of endothelial cells by decreasing NO production [42,43,44]. Furthermore, oxidative stress is a major contributor to the progression of CKD and CVD. ADMA increases oxidative stress and affects endothelial NO synthase coupling and NO bioavailability [45].

### 3.4. The Effect of ADMA on Kidney Injury

A significant relationship between blood pressure and plasma ADMA levels were observed in hypertensive patients [46]. Clinical data have also shown that plasma and serum ADMA levels were elevated with a decrease in the estimated GFR [47]. It has been reported that higher plasma ADMA levels are associated not only with mortality, but also with the progression to end-stage kidney disease (ESKD) [48,49]. However, regardless of normal kidney function, plasma ADMA levels are significantly correlated with proteinuria and serum levels of high-sensitivity C-reactive protein in patients with stage 1 CKD [50]. Furthermore, our previous study showed that serum ADMA levels were elevated in normotensive patients with chronic glomerulonephritis, as compared to healthy controls, and were significantly associated with arterial intimal fibroplastic thickness in the kidney [51]. 

Thus, an increase in circulating ADMA levels is also a first step in the process of the progression of CKD.

### 3.5. Role of pCS in the Progression of CKD

pCS also has adverse effects in the kidney, similar to those of IS. An experimental study using cultured proximal renal tubular cells showed that pCS, as well as IS, can induce significant cellular immune and inflammatory responses, including activation of the TGF-β signaling pathway [52]. Although the RAS and oxidative stress are important factors for exacerbation of CKD, pCS activates the RAS [53] and increases the amount of oxidative stress produced by stimulating leukocytes [54], which leads to renal tubular epithelial-to-mesenchymal transition. These changes contribute to the progression of kidney fibrosis. Klotho is a co-receptor for fibroblast growth factor-23. Besides this role, klotho is also related to the prevention of kidney fibrosis by modulating the TGF-β signaling pathway. Injection of pCS decreased klotho expression in renal tubules of mice and induced DNA hypermethylation [55]. Thus, pCS deteriorates kidney injury.

The previous clinical studies have also shown the adverse effects of pCS on CKD, as serum pCS levels were significantly associated with CKD progression, cardiovascular events, and mortality [56,57,58,59]. Previous studies have reported that AST-120 could decrease *p*-cresol and pCS [60]. However, thus far, no RCT has investigated the effect of AST-120 on serum pCS levels and correlations with clinical outcomes.

## 4. UTs and CVD

### 4.1. Pathophysiological Role of IS in CVD Progression

#### 4.1.1. IS and Cardiomyocytes

The role of IS in CKD was recently investigated. An important mechanism of IS is the induction of oxidative stress, which contributes to the progression of CVD. IS-induced oxidative stress hastens the progression of cardiac fibrosis and cardiac hypertrophy [61,62]. In fact, oxidative stress is significantly increased in patients with heart failure [63,64]. An in vitro study showed that the addition of IS increases the production of reactive oxygen species via an increase in the expression of NOX4 [22,65]. The study also demonstrated that IS increases the production of inflammatory cytokines, such as interleukin (IL)1-β, IL-6, and TNF-α [66].

As a direct effect of IS on cardiomyocytes, IS is taken up by cardiomyocytes through OAT-1 and -3, which leads to the subsequent activation of the NFκB, AMPK, and MAPK pathways [66,67]. The activation of these pathways induces the expressions of cardiac hypertrophy- and fibrosis-related molecules. In addition, the expression of klotho is decreased in CKD, which is related to cardiac hypertrophy and fibrosis [68]. Indeed, elevation of serum IS levels and impaired expression of klotho are reported to accelerate the progression of cardiac injuries [69].

#### 4.1.2. IS and Arrhythmia

It has been reported that atrial fibrillation (AF) is easily induced by atrial extrastimuli in hearts extracted from CKD model rats [70]. It has been suggested that IS could be a predisposing factor for AF in CKD mediated by the progression of atrial remodeling involving oxidative stress, inflammation, and fibrosis [70,71,72]. In addition to the progression of cardiac fibrosis, IS inhibits the function of inward rectifier potassium ion channels [73]. This inhibition may result in a prolonged QT interval.

#### 4.1.3. IS and the Vascular System

IS has several effects on the vascular system, including inhibiting the proliferation of endothelial cells and increasing the expression of adhesion molecules such as ICAM-1, VCAM-1, MCP-1, and e-selectin, which are involved in the pathophysiology and progression of atherosclerosis [74,75]. IS also induces the proliferation of vascular smooth muscle cells (VSMCs) by dose-dependent activation of P44/42 MAPK, which leads to the progression of atherosclerosis [76].

Although platelet aggregation is a crucial mechanism of vascular events, IS induces platelet aggregation and thrombus formation [77]. EPC is thought to be important for repair of vascular damage. As mentioned in the previous section, IS decreases the number and function of EPCs [78,79]. In CKD models of mice, IS was shown to impair neovascularization in ischemic hindlimbs, while lowering serum IS levels using AST-120 could improve this condition [80].

Furthermore, vascular calcification is an independent and important risk factor for the development of CKD and CVD. IS not only increases oxidative stress, but also increases the expression of bone-related proteins [81,82]. Through these pathophysiological mechanisms, IS seems to enhance transformation of aortic VSMCs into osteoblastic-like cells. Although phosphate is a key player in the progression of vascular calcification in CKD, vascular calcification is also promoted by IS.

### 4.2. The Association between IS and CVD

Several studies have investigated the association between IS and CVD. Higher serum IS levels are associated with increased CVD events [27], and patients with a history of CVD have higher serum IS concentrations, as compared to those without. Our previous study demonstrated that administration of AST-120 to patients with stage 4 or 5 CKD might prevent the progression of cardiac hypertrophy [83], which seems to support the results of experimental studies. The result of a previous clinical study on patients with chronic heart failure showed that higher serum IS levels were significantly associated with an increased prevalence of cardiac diastolic dysfunction, despite preserved kidney function [84]. Another study revealed that, independent of kidney function, serum IS levels were significantly associated with CVD events in patients with dilated cardiomyopathy [85]. Serum IS levels have also been correlated with prolonged QT intervals in CKD patients [73]. Even in dialysis patients, serum IS levels are associated with CVD, including heart failure [27,86] and peripheral artery disease [87]. In addition, serum IS levels have been associated with the severity of coronary artery disease [88] and restenosis after percutaneous coronary intervention [89]. There is a possibility that administration of AST-120 might prevent the progression of aortic calcification in patients with stage 4 or 5 CKD [90]. However, results of some recent studies suggest that IS is not associated with cardiovascular outcomes [91,92,93,94]. Therefore, close attention is required to verify the effect of AST-120 on CVD.

#### 4.2.1. Pathophysiological Roles of ADMA in the Progression of CVD

The major role of AMDA is decreasing NO through inhibition of NO synthase. NO has various effects on the cardiovascular system, such as regulation of blood pressure and vascular tone, inhibition of platelet aggregation and leukocyte adhesion, and prevention of smooth muscle cell proliferation [95]. It is well known that oxidized low-density lipoprotein (LDL) can drive the progression of atherosclerosis [96]. An in vitro study showed that incubation of endothelial cells with oxidized LDL, increased ADMA concentrations in the culture medium [97], and that the addition of ADMA to endothelial cells up-regulated lection-like oxidized LDL receptor-1 (LOX-1), which is the main oxidized LDL receptor in endothelial cells [98]. Oxidized LDL binds to the LOX-1 receptor and increases the intracellular generation of reactive oxygen species. An in vivo study also showed that long-term infusion of ADMA leads to increases in angiotensin-converting enzyme levels, oxidative stress, and coronary microvascular lesions [99]. Furthermore, ADMA activates the local RAS, and the release of angiotensin II activates NAD(P)H oxidase [100].

**Figure 3:**
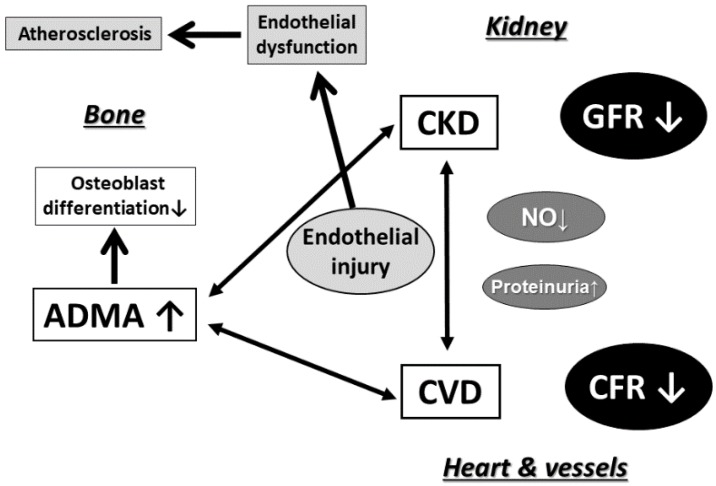
Putative role of ADMA in the kidney-cardiovascular-bone axis.

Thus, ADMA accelerates the progression of atherosclerosis and elevates blood pressure. These pathophysiological effects of ADMA on the vascular system contribute to the development of CVD (Figure 3). Hypertension itself is a crucial classical risk factor for CVD. Although hypertension and advanced atherosclerosis are involved in the mechanisms underlying the progression of CVD, the details of other mechanisms of ADMA in the progression of CVD remains unknown.

#### 4.2.2. The Association between ADMA and CVD

Many clinical studies have investigated the association between ADMA and CVD. We previously reported that plasma ADMA levels were significantly associated with impaired forearm arterial vascular function and impaired coronary flow reserve in hypertensive patients [47,101]. In addition, plasma ADMA levels were significantly associated with carotid intima-media thickness (IMT) in patients with CKD, as well as the general population [46,102,103]. Indeed, plasma ADMA levels were significantly associated with changes in IMT at follow-up [102]. Furthermore, a recent study reported that serum ADMA levels were significantly correlated with the presence and severity of coronary artery disease [104]. Taken together, ADMA is thought to cause endothelial dysfunction which is the first step in the progression of atherosclerosis.

A strong association between circulating ADMA levels and chronic heart failure has been confirmed by many clinical studies [105,106,107]. ADMA has been linked to various risk factors for heart failure. Accumulation of ADMA may cause an elevation of blood pressure, increase in afterload, impairment of myocardial blood flow, left ventricular hypertrophy, cardiac dysfunction, and the onset of atrial fibrillation [108,109,110,111]. Thus, ADMA contributes to the development of heart failure. In addition, it has been reported that systemic ADMA infusion decreases cardiac output and renal blood flow in a dose-dependent manner in healthy people [112]. The results of a meta-analysis of 30 studies comparing high versus low ADMA concentrations demonstrated that the relative risks for all-cause mortality was 1.52 (range, 1.37–1.68) and 1.33 (1.22–1.45) for CVD [113]. Considering these results, there is no doubt that there is a significant association between ADMA and CVD.

### 4.3. Role of pCS in the Progression of CVD

The effects of pCS on the progression of CVD have recently been clarified, and various mechanisms have been proposed based on the results of experimental and clinical studies [8,114]. An experimental study using human umbilical vein endothelial cells demonstrated that pCS increased the shedding of endothelial microparticles, which reflects endothelial damage [115]. Another effect of pCS on the vascular system is that pCS has been shown to induce oxidative stress, and to enhance vasoconstriction and vascular remodeling [116]. Furthermore, in ApoE (−/−) mice that underwent 5/6 nephrectomies, pCS promoted the progression of atherosclerotic lesions and disturbed the stability of formed plaques, probably by targeting VSMCs [117,118]. The results of the study showed that pCS facilitated the migration and proliferation of VSMCs and disrupted the balance between matrix metalloproteinases and tissue inhibitor of metalloproteinases within the plaques. As the detailed molecular mechanisms regarding the progression of vascular lesion, pCS also increased the expression of TNF-a, MCP-1, ICAM-1, VCAM-1, and E-selectin in a dose dependent manner [117].

The association between pCS and vascular calcification was also investigated in an in vitro study, which showed that pCS increased the expression of NOX4, alkaline phosphatase (ALP), osteopontin, and core-binding factor alpha 1 [119]. However, knockdown of NOX4 and the presence of probenecid, which is an inhibitor of OAT, suppressed the expression of these molecules, suggesting that pCS-induced oxidative stress by intracellular accumulation via OAT might promote vascular injury. In addition, although insulin resistance has been attributed to the progression of CVD, it is a well-documented feature of CKD. It has been reported that mice treated with pCS exhibited altered insulin signaling in skeletal muscles through ERK1/2 activation, which led to insulin resistance [120]. This mechanism is thought to be due to direct activation of ERK1/2 by pCS.

Previous reports have demonstrated that serum pCS levels were significantly correlated with the severity of coronary artery lesions, the presence of peripheral artery disease, and vascular access failure events [87,121]. Moreover, the results of a meta-analysis revealed that serum pCS levels were significantly associated with CVD events and all-cause mortality in hemodialysis patients [94,122,123]. In light of these results, pCS could be used as a predictive marker of CVD and mortality in the clinical setting.

## 5. UTs and Bone Disease

Patients with CKD are at higher risk for fracture, compared to the general population [124]. CKD is associated with a wide spectrum of bone diseases caused by hyperparathyroidism and vitamin D deficiency. In addition, UTs are also associated with bone disease.

### 5.1. Association between IS and Bone Disease

Some studies have investigated the association between IS and bone diseases in patients with CKD. In vitro studies have demonstrated that IS inhibited differentiation of mesenchymal stem cells into osteoblasts, osteoblastic cell proliferation, bone mineralization, ALP activity, and the expression of bone formation-related genes [125,126,127,128]. Although the molecular mechanisms that underlie the inhibition of differentiation are unclear, oxidative stress induced by uptake of IS into osteoblastic cells via OAT-3 is thought to be one of the crucial mechanisms [125,126,127]. Oxidative stress is increased depending on the extracellular IS concentration. The OAT inhibitor probenecid decreases intracellular IS concentrations, suppresses IS-induced free radical production, and improves osteoblastic cell viability. In addition, the antioxidant, *N*-acetylcysteine can also improve osteoblastic cell viability.

Although many patients with ESKD manifest hyperparathyroidism, the action of parathyroid hormone (PTH) is blunted in uremic status. Therefore, these patients are thought to have skeletal resistance to PTH [129]. This resistance is associated with many factors, including UTs. IS was shown to decrease PTH-induced intracellular production of cyclic adenosine monophosphate (cAMP) and PTH receptor gene expression in primary osteoblastic cell cultures [125].

An association between IS and osteoclasts was reported in two studies [127,130]. IS inhibited the receptor activator of nuclear factor kappa B ligand-dependent osteoclast differentiation and reduced bone-resorbing activity of osteoclasts in a dose-dependent manner. Osteoclasts also express OAT-3 and probenecid blocks IS-induced osteoclast differentiation.

Few in vivo studies have investigated the effect of IS on bone. A thyroparathyroidectomized and nephrectomized rat is an animal model of low turnover bone in uremic status [131]. In this model, it has been reported that AST-120, an oral charcoal adsorbent of UTs including IS, decreased serum IS levels and suppressed the progression of low turnover bone, as assessed by histomorphometry. Expression of ALP, osteocalcin, and PTH receptor genes were also ameliorated [132]. Moreover, AST-120 may improve bone chemical composition, including pentosidine, in this rat model [133]. Pentosidine is one of the advanced glycation end products and its accumulation in bones induces bone fragility [134]. AST-120 suppresses the accumulation of pentosidine in cortical bone [133]. Another study examined the effect of IS on bone in non-CKD rats that underwent only parathyroidectomy [135] and found a decrease in bone formation-related parameters in parathyroidectomized rats by administering indole, which is a precursor of IS, while bone resorption–related parameters were unchanged. Despite the administration of either AST-120 or indole, bone mineral density did not significantly change.

A cross-sectional study of 47 hemodialysis patients found that IS was negatively correlated with bone formation markers, such as ALP and bone-specific ALP, independent of intact PTH levels [136]. This result suggests that high serum IS levels may be associated with low bone formation in humans, in accordance with the results of basic studies. Another study demonstrated that serum IS levels were positively correlated with serum sclerostin levels in 154 patients with stages 2-5D CKD [137]. Sclerostin is a circulating inhibitor of the Wnt/β-catenin pathway and high serum sclerostin levels are associated with a greater risk of fracture [138]. However, the interactions between IS and sclerostin remain unclear.

### 5.2. Association between ADMA and Bone Disease

Although ADMA is an inhibitor of NO synthase, it may also act on osteoblasts. A previous experimental study examined the effect of ADMA on the differentiation of bone marrow-derived mesenchymal stem cells into osteoblasts [139]. In this study, ADMA was associated with a reduction in ALP activity, calcium deposition, and osteoblast-related gene expression. Another study also showed that ADMA inhibited osteoblastic differentiation [128]. Thus, although there is little evidence regarding the influence of ADMA on bone disease, ADMA may inhibit the differentiation of osteoblasts.

### 5.3. Association between pCS and Bone Disease

pCS may induce osteoblastic dysfunction. In vitro studies have shown that pCS inhibited osteoblastic differentiation, cell viability, cell proliferation, PTH-induced intracellular production of cAMP, and PTH receptor gene expression [128,140]. Interestingly, pCS inhibited the function of osteoblasts at even lower concentrations at which pCS did not increase oxidative stress [140]. Alternatively, the study also demonstrated that activation of JNK/p38 MAPKs was associated with pCS-induced osteoblastic dysfunction. This mechanism is different from the effect of IS.

Few animal and clinical studies have investigated the effect of pCS on bone. However, since AST-120 absorbs not only IS, but also pCS, the effect of AST-120 on bone may be partially due to pCS. In fact, our previous clinical study demonstrated that serum pCS levels were positively correlated with serum sclerostin levels [137]. Therefore, high pCS levels may also be associated with fracture.

## 6. Conclusions

Although the term “uremia” is often used, it merely describes various vague conditions observed in patients with ESKD. Recently, numerous UTs have been identified owing to scientific developments [141], and the detailed mechanisms of vicious influences on the human body have also been clarified. However, of course, there may be a huge number of UTs that have not yet been identified, and some pathophysiological mechanisms of known UTs may be unknown. Further detailed investigations to clarify the role of UTs and to develop useful therapeutic strategies to prevent the progression of CKD, CVD, and bone disease are warranted.

## Figures and Tables

**Table 1:** Classification of major uremic toxins.

Small Water-Soluble Compounds (<500 Da)	Middle Molecule (≥500 Da)	Protein Bound Compounds (Mostly < 500 Da)
ADMA	ANP	AGEs
Carbamylated compounds	β_2_-microglobulin	Homocysteine
Creatinine	Endothelin	Indoxyl sulfate
SDMA	FGF23	Indole acetic acid
TMAO	Ghrelin	Kynurenines
Urea	Immunoglobulin light chains	*p*-cresylsulfate
Uric acid	Interleukin-6	Phenyl acetic acid
	Interleukin-8	
	Interleukin-18	
	Lipids and lipoproteins	
	Neuropeptide Y	
	PTH	
	Retinol binding protein	
	TNF-α	

ADMA, asymmetric dimethylarginine; SDMA, symmetric dimethylarginine; TMAO, trimethylamine-*N*-oxide; ANP, atrial natriuretic peptide; FGF23, fibroblast growth factor 23; PTH, parathyroid hormone; TNF-α, tumor necrosis factor-α, AGEs, advanced glycation end products.
